# From Novel Technology to Novel Applications: Comment on “An Integrated Brain-Machine Interface Platform With Thousands of Channels” by Elon Musk and Neuralink

**DOI:** 10.2196/16356

**Published:** 2019-10-31

**Authors:** Alexander N Pisarchik, Vladimir A Maksimenko, Alexander E Hramov

**Affiliations:** 1 Center for Biomedical Technology Technical University of Madrid Pozuelo de Alarcón, Madrid Spain; 2 Neuroscience and Cognitive Technology Laboratory Center for Technologies in Robotics and Mechatronics Components Innopolis University Innopolis Russian Federation

**Keywords:** brain-computer interface, brain-machine interface, brain, electroencephalography

The first attempts to translate neuronal activity into commands to control external devices were made in monkeys yet in 1960s [1]. After that, during 1960-1970, the biological feedback was realized in monkeys, to provide voluntary control of the firing rate of cortical neurons [2,3]. The term “brain-computer interface” appeared only in earlier 1970s [4]. The brain-computer interface is usually referred to as a “brain-machine interface” in invasive studies. Nowadays, the brain-computer interface and brain-machine interface research and applications are considered one of the most exciting interdisciplinary areas of science and technology.

In particular, brain-computer interfaces are very promising for neurorehabilitation of sensory and motor disabilities [5], neurocommunication [6], exoskeletons [7], cognitive state evaluation [8], etc. Advanced mathematical methods for extraction and classification of neuronal activity features hold out hope for the future use of brain-computer interfaces in everyday life. At the same time, the lack of effective invasive neuroimaging techniques providing a high-resolution neural activity recording for medical purposes limits the brain-machine interface implementation in clinics.

In their paper, Elon Musk and Neuralink [9] have successfully addressed the major issues hampering the next generation of invasive brain-computer interface (or brain-machine interface) development by introducing a novel integrated platform enabling a high-quality registration of thousands of channels. Their device contains arrays of flexible electrode threads with up to 3072 electrodes per array, distributed across 96 threads. To overcome a surgical limitation, the authors have built a neurosurgical robot that inserts 6 threads per minute with a micrometer spatial precision. To increase the biocompatibility, they created a neurosurgical robot, which implants polymer probes much faster and more safely than existing surgical approaches. Using this platform in freely moving rats, the authors report a spiking yield of up to 85.5%.

Although the developed system is considered an effective platform for research in rodents, it can serve as an invasive neurointerface prototype for clinical applications. Specifically, multielectrode neurointerfaces may become the basis for new communication systems and advanced assistive technologies for paralyzed people as well as control external devices and interact with the entire environment, eg, by integrating into new fast developed technologies, such as Smart Home and Internet of Things. Moreover, the brain-computer interface applications are very promising for detecting hidden information in the user’s brain, which cannot be revealed by conventional communication channels. Currently, the use of noninvasive brain-computer interfaces in these fields is limited by a low number of commands that can be recognized. This limitation arises from a relatively small number of features, which can be extracted from the scalp-level electroencephalography or functional near-infrared spectroscopy recordings. The invasive brain-computer interfaces (or brain-machine interfaces) demonstrate much better performance than noninvasive brain-computer interfaces; however, they require a larger number of channels to obtain more detailed information about the individual spiking activity of the neurons across distributed cortical regions. The device reported in the paper of Elon Musk and Neuralink approaches the solution to this problem.

One of the most promising applications of noninvasive brain-computer interfaces is monitoring, control, and training of human’s psychophysiological states and cognitive abilities. In such studies, the subject’s mental state is continuously evaluated by the passive brain-computer interface. The passive brain-computer interface analyzes the current brain activity of the user without any aim to control command generation and provides information about features of the actual brain activity related to attention, emotional state, fatigue, etc [10]. These top-down processes originating on the cortical level are spatially distributed and relatively slow, and therefore, they have well-pronounced markers on the noninvasive electroencephalography and functional near-infrared spectroscopy signals.

However, in the case of spatially localized neuronal activity, the noninvasive techniques are unable to recognize distinguished features of neurophysiological diseases in real time, and therefore, one needs to insert electrodes into particular brain regions. For example, to predict epileptic seizures, the neuronal activity must be recorded from predefined focal areas of the brain, where an earlier manifestation of this pathological activity is mostly pronounced and can be promptly detected in real time [11]. Recently, an efficient method for epilepsy prediction based on electrical brain activity was proposed [12,13]. The method allows forecasting a focal seizure up to 5 seconds before it occurs. This time is sufficient for a brain-machine interface to generate a proper signal, which suppresses the forthcoming seizure. For drug-resistant patients, the complete abolishment of epileptic seizures might be achieved by a brain-machine interface/brain-computer interface that predicts a seizure onset, combined with a system that interferes with the process that causes the seizure. Thus, seizure prediction remains an unresolved problem due to insufficient information about neural processes in the onset brain area. Therefore, one possible and very important clinical application of the Neuralink technology is a brain-machine interface for patients with drug-resistant epilepsy. These brain-machine interfaces should imply the brain stimulation (electrical, magnetic, optogenetic, etc) to interrupt or even prevent epileptic seizures. The stimulation can be delivered to the brain in either an open-loop or a closed-loop fashion. In the former case, there is no need to monitor the current brain activity, since the stimulation is activated manually or in accordance with a predefined stimulation protocol.

**Figure 1:**
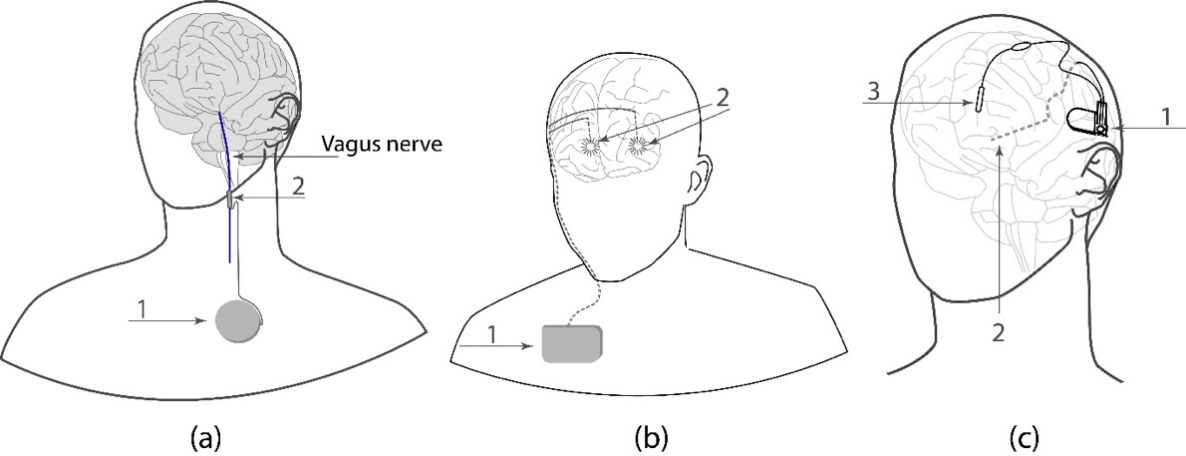
The schematic illustration of brain-machine interface prototypes to suppress epileptic seizures using electrical stimulation. (a) Vagus nerve stimulator containing (1) an implantable pulse generator and (2) a stimulation lead. (b) Stimulator of the anterior nucleus of the thalamus in epilepsy containing (1) an implantable pulse generator and (2) intracranial electrodes placed in the anterior thalamic nuclei bilaterally. (c) Responsive neurostimulator containing (1) implanted deep electrodes for recording electroencephalography signals, (2) an implantable device for processing electroencephalography signals from electrodes, and (3) strip electrodes receiving an electrical stimulation signal generated by the device to stop seizures.

As an example of the open-loop system, in Figure 1a,we present the vagus nerve stimulator manufactured by Cyberonics, Inc (Texas, United States) in 1977. The stimulator contains an implantable pulse generator and an electrode to stimulate the left vagus nerve in a repetitive “duty cycle” (“on” for 30 seconds and then “off” for 5 minutes), which allows reducing the number of seizures by an average of 30%–40% [14]. Along with the vagus nerve stimulator, other open-loop antiepileptic devices are also used for deep brain stimulation. One of the first open-loop deep brain stimulators was the stimulator of the anterior nucleus of the thalamus in epilepsy proposed by Medtronic, Inc (United States) for patients with partial-onset epilepsy [15] (Figure 1b).

Although the open-loop systems enable a significant reduction in the number of epileptic seizures, it is obvious that a more efficient control of epileptic activity requires continuous monitoring of the current brain activity, which can be achieved by using closed-loop brain-machine interfaces. One of the first closed-loop brain-machine interfaces was the responsive neurostimulator designed by Neuropace Inc. (California, United States; Figure 1c). It contains implanted electrodes for recording the intracranial electroencephalography used by the algorithm, which determines the moment of time when a seizure starts. To interrupt the seizure, the triggered focal electrical stimulation is sent to a specific brain area [16].

**Figure 2:**
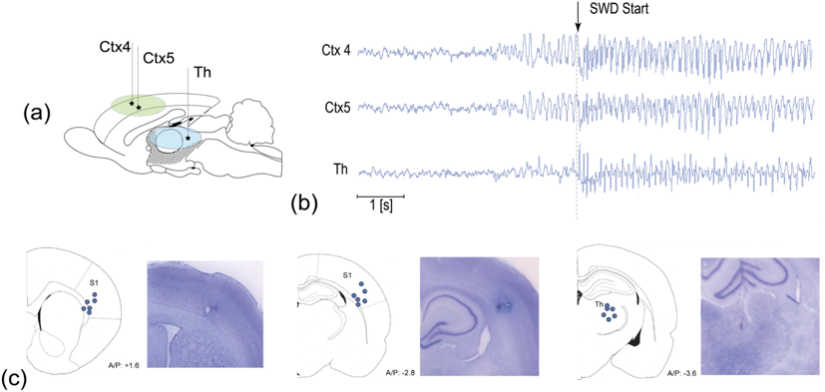
(a) Schematic representation of the experiments with a rat. (b) The set of electrocorticography recordings taken from subgranular layers 4 (Ctx4) and 5 (Ctx5) of the somatosensory cortex and postero/lateral thalamus before and during onset of the epileptic spike-wave discharge. (c) Histological verification of the electrode location in the somatosensory cortex (S1) and postero/lateral thalamus (based on data from [<xref ref-type="bibr" rid="ref11">11</xref>]). Th: thalamus.

It is important to point out that epileptic seizures are well detected using electrocorticography or intracranial electroencephalography, which display a pronounced marker of the high-amplitude rhythmic activity. Recent studies reported a 100% accuracy of this technique in detecting epileptic seizures in rats [17]. Since the preictal activity may not differ from a normal behavior, the prediction of seizures is a very challenging task. Although existing algorithms allow seizure predictions with high sensitivity, they are too complicated and too specific to be used in clinics [18]. One of the closed-loop systems for the seizure prediction and prevention was recently tested in vivo in rats [11] (Figure 2). The seizure prediction algorithm was based on the electrocorticography signals recorded by three electrodes in the cortex and the thalamus, as shown in Figure 2a. The brain-machine interface was able to correctly predict 45% seizures, but the number of false predictions varied from 20 to 100 per hour among animals. In this regard, one can expect that the technique developed by Elon Musk and Neuralink with thousands of channels will significantly improve seizure prediction. The large number of registered channels will increase classification accuracy in pre-epileptic state recognition and decrease the number of false positives during light slow wave sleep. This is indeed a step toward the next generation of brain-machine interfaces for drug-resistant epilepsy. The accurate seizure prediction algorithm based on the multichannel Neuralink technology enables the prevention of ongoing seizures and protects the patient against unnecessary stimulations caused by false alarms. Thanks to the developed brain-machine interface platform with thousands of channels, significant progress is expected in solving this important problem, enabling new clinical trials for patients resistant to drug therapy.

Another type of brain dynamics associated with spatially localized cortical sources is a motor-related neuronal activity. This problem has a significant social impact. Today, most brain-computer interfaces and brain-machine interfaces are designed for patients with severe motor disorders. The brain-computer interface allows a disabled person to control wheelchairs, exoskeletons, and robotic manipulators by generating commands via voluntary changes of brain activity in the motor cortex induced by motor imagery. For instance, these brain-computer interfaces are used to control a cursor or a wheelchair in two dimensions with the intention of moving the left or right hand [19]. However, the performance of such noninvasive brain-computer interfaces for movement control is limited by a small number of commands. Studies on animals, primarily monkeys, have shown the effectiveness of invasive registration of cortical neuron activity to create an effective brain-machine interface for controlling more complex movements. In 2008, an invasive interface was implemented, which allows a monkey to control an anthropomorphic manipulator [20]. In the experiment, the signal from 15-25 cortical units was recorded in the monkey, to control a robotic arm to feed itself. The monkey performed a continuous self-feeding task with a mean success rate of 69.5%. Similar results were obtained with an invasive interface that controlled the lower limbs [21] or both hands simultaneously (bimanual movements) [22] using cortical activity patterns.

It should be noted that the use of invasive brain-machine interfaces allows patients with tetraplegia to perform reach and grasp tasks with a robotic arm manipulator [23]. At the same time, the brain-machine interface implementation in humans is still not employed in clinical practice due to surgical difficulties and the problems of biocompatibility. In this context, the proposed neurosurgical robot can be considered an important step toward human implants. A significant advantage of the robot is its rapid manipulation to insert six-electrode threads per minute in addition to its capacity in precisely inserting the most biocompatible polymer probes. Another potential clinical application of the brain-machine interface might be the restoration of neuronal connections, lost due to degenerative diseases, such as Alzheimer disease, or replacement of dead neurons with artificial ones. Comprehensive studies covering many aspects of this issue are currently underway [24].

Finally, we would like to draw attention to the importance of the neural activity modulation in the next-generation brain-machine interfaces. This possibility is essential for applications such as neuroprosthetics in order to provide biological feedback as a sense of touch [25], and new therapeutic approaches for patients with drug-resistant epilepsy in order to prevent ongoing seizures by the appropriate electric stimulation [26]. In view of the foregoing, the authors of the paper highlight the capability of their custom electronics to deliver an electrical stimulation to every channel, but unfortunately, do not present these results in the paper. It would be interesting to know how the authors plan to deliver electrical pulses to cells and record the neural activity simultaneously, in particular, whether the stimulation preserves the potential for simultaneous recording of the neural activity with the minimized effect of stimulus-induced artifacts. If this issue is successfully addressed, it would become possible to interact with the neural activity in the continuous manner instead of the neuron stimulation with the electrical pulses in a discrete closed-loop fashion. Nowadays, this is achieved by the optogenetic brain stimulation [27,28] with the help of hybrid optoelectronic interfaces [29] and thought to be a substantial advantage of optogenetics over the electrical stimulation and recording.

One of the main obstacles for clinical applications of implanted devices is their low biocompatibility that does not allow long-term recordings. Elon Musk and Neuralink approach this problem by utilizing a biocompatible polyimide, which encapsulates a gold thin film trace. By choosing electrically conducted materials, one has to play between impedance and biocompatibility. The authors tested the polyethylenedioxythiophene-doped polymer with polystyrene sulfonate and iridium oxide and achieved lower impedance for the former, but better biocompatibility for the latter. They promise to continue research in this direction and extend their techniques and processes to other types of conductive electrode materials and coatings.

Last, but not the least, are the unpleasant consequences of this work that we should pay attention to, since every achievement of humanity has two sides. On one hand, it intends to improve the quality of life, but on the other hand, it can be used by unscrupulous people for their selfish goals. Therefore, every scientist should think not only about a positive impact of his/her research, but also about its possible negative effects. Among the undesired effects of brain-machine interfaces with electrodes implanted into the human brain is the possibility that a government or a nongovernmental organization will control and manipulate the person’s behavior not only through mass media, but also by directly sending commands to the brain. In this regard, numerous debates about ethics of using brain-machine interfaces are currently underway in the media.

In summary, the novel neurointerface by Elon Musk and Neurolink has all chances to become a real step forward to the next generation of brain-machine interfaces for both research and clinical applications. Invasive interfaces can help disabled people control external devices and communicate with other people. We believe that future communication technologies will be based on brain-computer interfaces that will read brain signals and translate them to messages, which then will be sent to mobile or other devices. Furthermore, invasive brain-machine interfaces will allow a direct communication between people by their thoughts.
